# Dermoscopy of spiradenocylindroma

**DOI:** 10.1016/j.jdcr.2025.03.003

**Published:** 2025-03-24

**Authors:** Susan Pei, Drew Kuraitis

**Affiliations:** a Department of Dermatology, Roswell Park Comprehensive Cancer Center, Buffalo, New York; b Department of Dermatology, Tulane University, New Orleans, Louisiana

**Keywords:** adnexal, basal cell carcinoma, blood vessels, dermoscopy, spiradenocylindroma

## Clinical presentation

**Fig 1:**
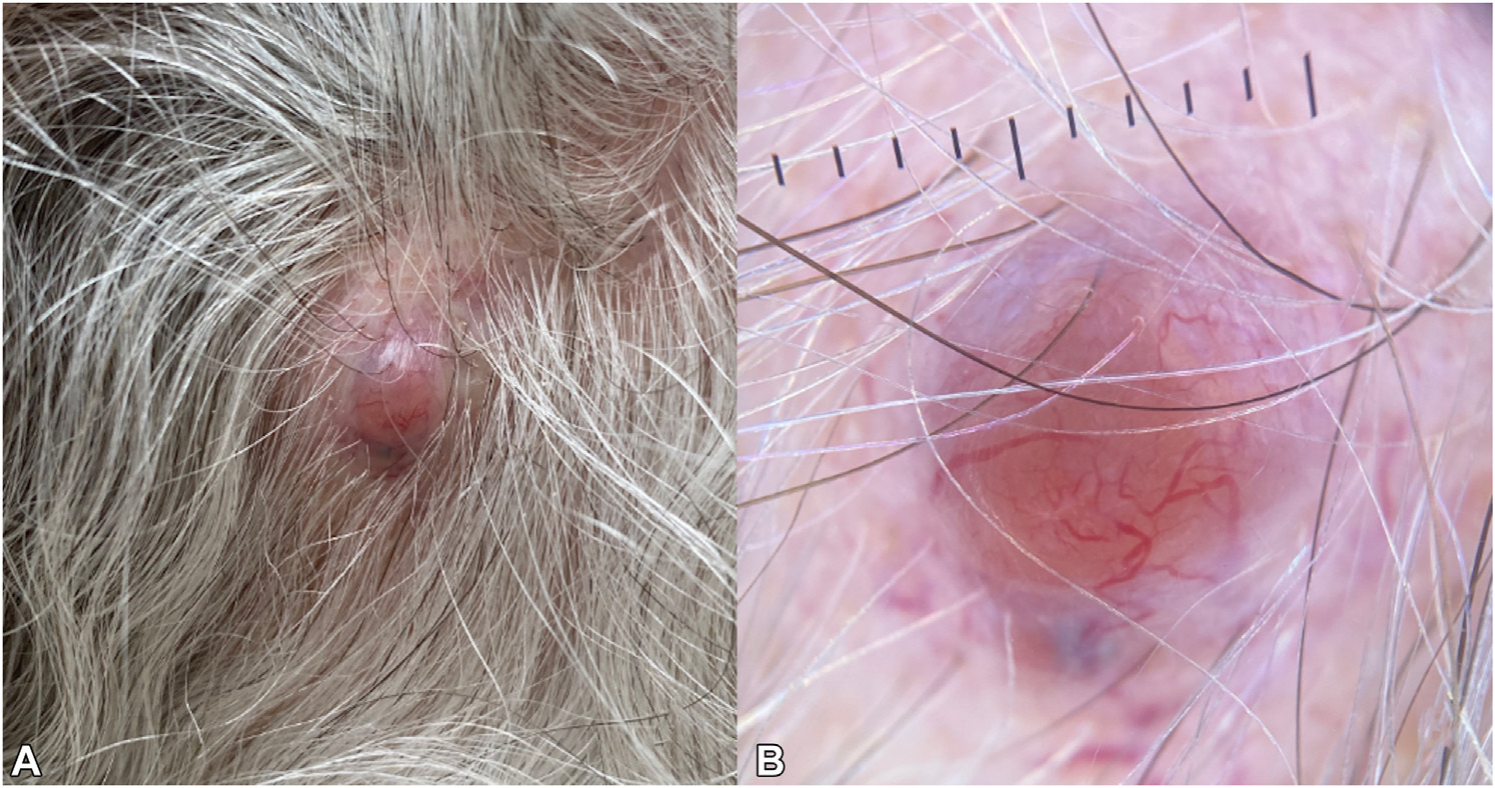
*Pink* papule on the scalp with prominent vasculature visible without further magnification (**A**). Dermatoscopy of spiradenocylindroma demonstrating a homogeneous salmon or *pink* background with superficial angulated and tortuous vessels (**B**).

A 72-year-old man with a history of numerous basal cell carcinomas (BCCs) on the head and neck presented to dermatology for evaluation of a new lesion. The scalp had a 7 mm firm, tender papule that had reportedly grown within 1-2 months (Fig 1, *A*).

## Dermatoscopic appearance

Dermatoscopy revealed numerous angulated and tortuous vessels on a homogeneous pink background with rare shiny white lines but an absence of prominent pigment and leaf-like structures (Fig 1, *B*).

## Histologic diagnosis

**Fig 2:**
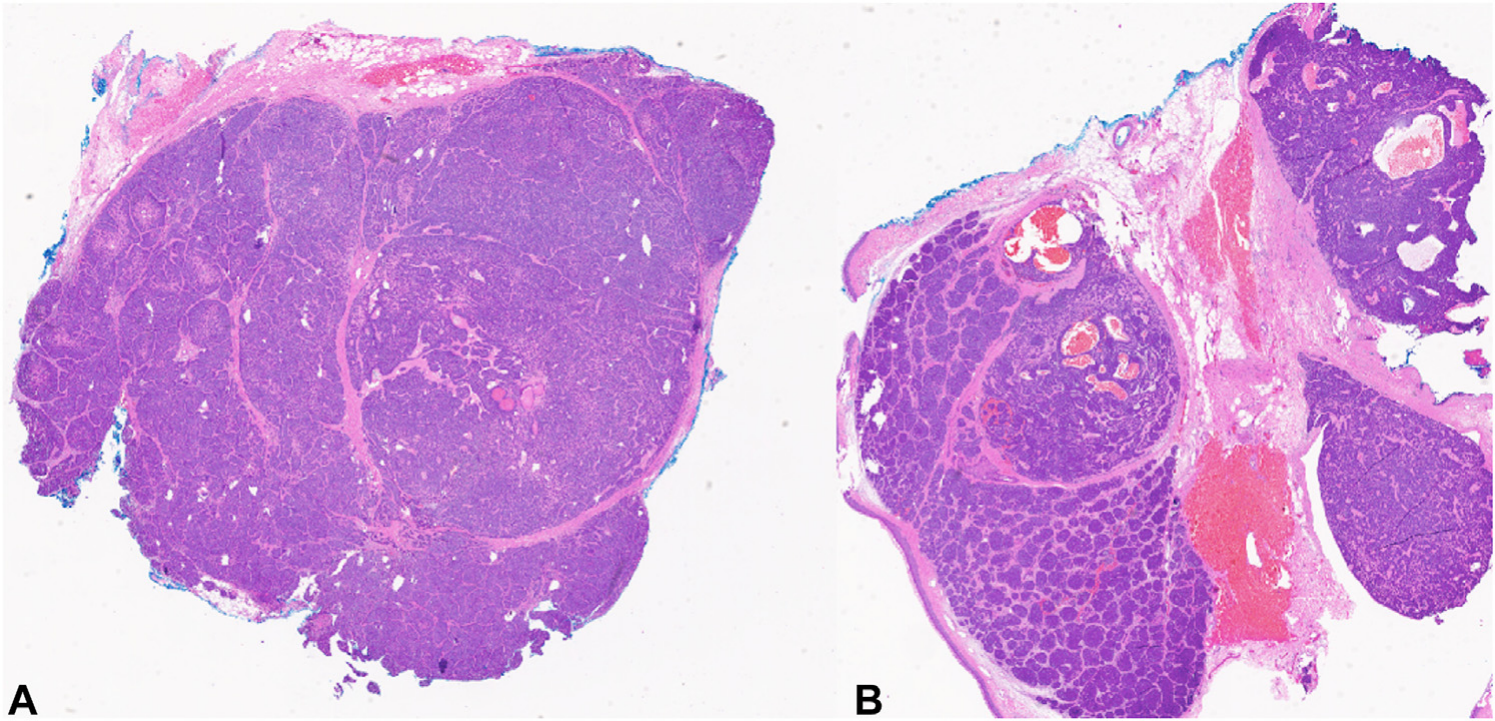
On histopathology, there is a circumscribed, dermal, multinodular basophilic tumor at different levels (**A** and **B**, hematoxylin and eosin, 15×). Tumor nodules are comprised of larger pale cells centrally with smaller basaloid peripheral cells with globules of eosinophilic hyaline material.

**Fig 3:**
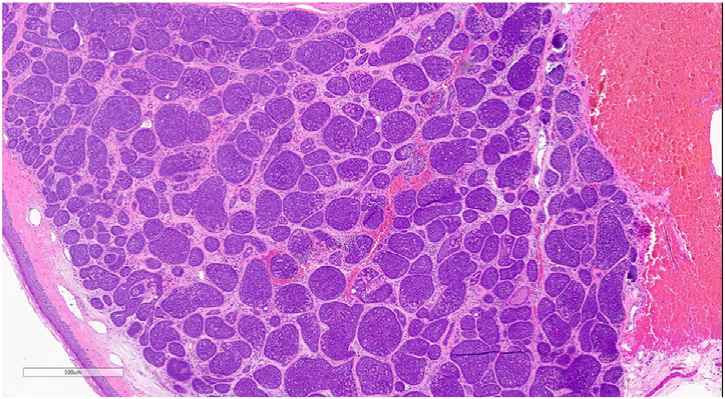
Focally, some tumor islands are arranged in a “jigsaw pattern,” surrounded by eosinophilic basement membrane (hematoxylin and eosin, 50×).

Histopathologic examination demonstrated a well-circumscribed, dermal, multinodular basophilic tumor comprised of larger pale cells centrally with smaller basaloid peripheral cells (Fig 2), with intratumoral lymphocytes and ductal structures with focal areas of basaloid tumor lobules demonstrating a “jigsaw pattern” arrangement (Fig 3), consistent with a hybrid spiradenocylindroma.

**Key message**

Spiradenomas and cylindromas are related benign adnexal tumors with eccrine differentiation, commonly arising on the head. Rarely, a hybrid of both tumors presents, referred to as a spiradenocylindroma. Adnexal tumors commonly mimic BCC on dermatoscopy,^1^ presumably due to the frequency of arborizing vessels, which is a characteristic feature of BCC^2^; however, dermatoscopy of a hybrid spiradenocylindroma has rarely been reported. The lesion presented demonstrated arborizing and tortuous vessels, leading to the inclusion of BCC in the differential diagnosis, although it was unusually tender. This case further expands on the dermatoscopic findings of a rare adnexal tumor.

## Conflicts of interest

None disclosed.
